# Antioxidant Phenolic Compounds of *Dracaena Cambodiana*

**DOI:** 10.3390/molecules15128904

**Published:** 2010-12-06

**Authors:** Ying Luo, Hui Wang, Xuerong Xu, Wenli Mei, Haofu Dai

**Affiliations:** 1 Key Laboratory of Tropical Crop Biotechnology, Ministry of Agriculture, Institute of Tropical Bioscience and Biotechnology, Chinese Academy of Tropical Agricultural Sciences, Haikou 571101, China; 2 Hainan Key Laboratory for Research and Development of Natural Product from Li Folk Medicine, Haikou 571101, China

**Keywords:** *Dracaena cambodiana*, DPPH, ABTS^+^, antioxidant activity, chemical constituent

## Abstract

The antioxidant activities of the petroleum ether, ethyl acetate, *n*-BuOH and water extract fractions from *Dracaena cambodiana* Pierre ex Gagnep were evaluated in this study. The ethyl acetate fraction contained the highest amount of total phenolics and total flavonoids, and showed the greatest DPPH˙, ABTS^+^ and Superoxide anion radical-scavenging capacities. The DPPH˙, ABTS^+^ and Superoxide anion radical-scavenging capacities of nine compounds isolated from the ethyl acetate fraction were also evaluated. The results indicated that these compounds contributed to the antioxidant activity of *D. cambodiana*. Therefore, *D. cambodiana* and these compounds might be used as natural antioxidants.

## 1. Introduction

In recent years, there has been an increasing volume of evidence to suggest that many age-related human diseases such as heart disease, cancer, arthritis, immune system decline, brain dysfunction, and cataracts are the results of cellular damage by free radicals, and that antioxidants in our diet could play an important role in the prevention of such diseases [1,2]. This has attracted much public interest in natural antioxidants and has led to an extensive search for effective antioxidants in nature [3,4,5,6,7]. At the same time, numerous crude extracts and pure natural compounds from natural plants have been reported to possess antioxidant and radical-scavenging capacities. For example, flavonoids, with a large distribution in nature, have strong antioxidant activity [8,9].

**Figure 1:**
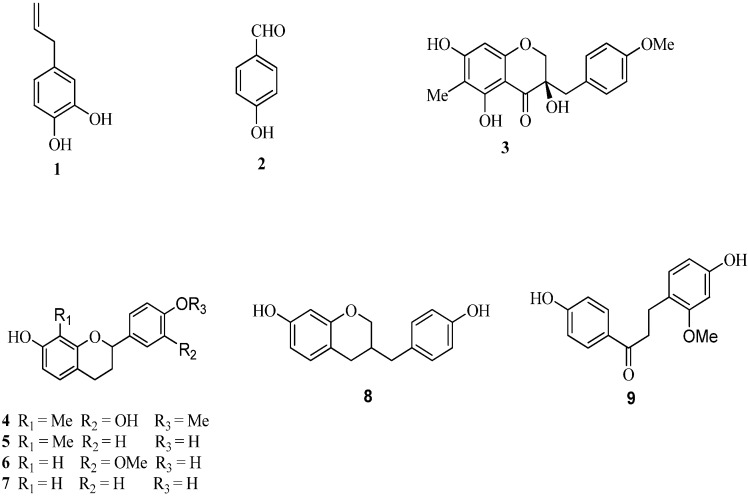
Structures of compounds **1**–**9**.

Dragon’s blood is the red resin secreted by the stems of *Dracaena*, *Daemonorops*, *Croton,* and *Pterocarpus* [10]. As a famous traditional medicine in the ancient Arab culture, dragon’s blood has been used for the treatment of wounds, leucorrhea, fractures, diarrhea, and piles as well as intestinal and stomach ulcers for a long time [11]. In China, dragon’s blood is produced form *Dracaena cochinchinensis* (Lour.) and *Dracaena cambodiana* [12]. Previous phytochemical studies on the plants of the genus *Dracaena* have led to the isolation of a number of phenolic compounds and a series of steroidal saponins [13], which are the same or similar to those compounds discovered in dragon’s blood originated from plants of the genus. Nine compounds, namley 3,4-dihydroxyallylbenzene (**1**), *p*-hydroxybenzaldehyde (**2**), cambodianol (**3**), (2*S*)-3',7-dihydroxy-4'-methoxy-8-methylflavane (**4**), (2*R*)-4',7-dihydroxy-8-methylflavane (**5**), (±)-4',7-dihydroxy-3'-methoxyflavane (**6**), (2*S*)-4',7-dihydroxyflavane (**7**), 7,4'-dihydroxy-homoisoflavane (**8**) and 4,4'-dihydroxy-2'-methoxychalcone (**9**) (Figure 1) were isolated from the ethyl acetate fraction of the *D. cambodiana* stem extract [14,15,16]. Their structures are similar to those of compounds found in dragon’s blood obtained from *D. cochinchinensis*. In continuation of our work, this report deals with the antioxidant properties of fractions and phenolic compounds derived from the ethanol extract of the stem of *D. cambodiana* for use as natural antioxidants. 

## 2. Results and Discussion

### 2.1. Fractions

Table 1 shows the DPPH and ABTS radical-scavenging capacities of the fractions from *D. cambodiana*. The results illustrated that the DPPH and ABTS radical-scavenging capacities of various solvent fractions increased with increasing concentration, and all the regression equations were significant at p < 0.05, so all the fractions had some DPPH and ABTS free radical-scavenging capacities. The concentrations of the fractions (25–800 μg/mL) or individual pure compounds(6.25–200 μg/mL) were used in developing the respective standard curves.

**Table 1:** The DPPH and ABTS free radical-scavenging capacity (%) of the fractions from *D. cambodiana*.

Concentration μg/mL	DPPH radical-scavenging capacity (%)					ABTS free radical-scavenging capacity (%)				
	PEF	EAF	BUF	AF	Vc	PEF	EAF	BUF	AF	Trolox^®^
800	11.90	29.49	18.22	12.45	ND	28.79	69.10	42.42	35.96	ND
400	-	16.02	-	-	ND	17.56	39.47	25.43	23.46	ND
200	2.93	-	6.04	7.33	ND	12.08	23.03	15.73	17.98	99.58
100	1.60	4.30	3.30	2.66	73.22	7.86	14.61	10.39	8.43	65.45
50	0.64	3.94	2.84	1.74	35.61	5.62	10.39	7.44	7.16	38.34
25	0.18	2.66	0.73	1.65	19.56	4.78	9.41	6.60	5.06	16.43
12.5	ND	ND	ND	ND	5.41	ND	ND	ND	ND	10.39
6.25	ND	ND	ND	ND	4.71	ND	ND	ND	ND	6.60

DPPH radical-scavenging capacity: The linear equations of Vc and the extracts were shown as follows. Vc: y = 0.0074x − 0.0110 (R^2^ = 0.9958 **); PET: y = 0.0001x − 0.0007 (R^2^ = 0.9995 **); EAF: y = 0.0003x + 0.0162 (R^2^ = 0.9980 **); BUF: y = 0.0002x + 0.0118 (R^2^ = 0.9915 **); AF:y = 0.0001x + 0.0126 (R^2^ = 0.9966 **). ABTS free radical scavenging assay: The linear equations of Trolox^®^ and the extracts were shown as follows. Trolox^®^: y = 0.0050x + 0.0569 (R^2^ = 0.9749 **); PET: y = 0.0003x + 0.0470 (R^2^ = 0.9930 **); EAF: y = 0.0008x + 0.0718 (R^2^ = 0.9992 **); BUF: y = 0.0005x + 0.0578 (R^2^ = 0.9975 **); AF: y = 0.0004x + 0.0599 (R^2^ = 0.9596 **). The symbol ** followed with the determination coefficient shows the significant level at 0.01, and the symbol * followed with the determination coefficient shows the significant level at 0.05, and no symbol followed with the determination coefficient shows no significance. ND represents not done. “-” represents the data missed.

The DPPH (1,1-diphenyl-2-picrylhydrazyl) radical-scavenging assay is widely used to evaluate antioxidant capacity in a short time [17,18]. The DPPH radical-scavenging capacities of the fractions of *D. cambodiana* stems are shown in Table 2. Among the fractions, the EC_50_ values of the DPPH radical-scavenging capacities were found to be 5.007 mg/mL, 1.613 mg/mL, 2.441 mg/mL and 4.874 mg/mL for petroleum ether fraction (PEF), ethyl acetate fraction (EAF), *n*-butanol fraction (BUF), and aqueous fraction (AF), respectively. The order of DPPH radical-scavenging capacities was found to be EAF > BUF > AF > PEF. The EAF and BUF fractions showed the most potent activity, while the PEF was the least active scavenger, indicating that compounds with strongest radical-scavenging capacities were located in the medium polarity section.

Table 2 also illustrates the ABTS free radical scavenging capacity of the fractions of *D. cambodiana* stems. Among the fractions, the ABTS radical-scavenging capacity IC_50_ values were found to be1.510 mg/mL, 0.535 mg/mL, 0.884 mg/mL and 1.100 mg/mL for PEF, EAF, BUF and AF, respectively. The order of ABTS radical-scavenging capacities was found to be EAF > BUF > AF > PEF.

**Table 2:** The DPPH˙, ABTS^+^ and Superoxide anion radical-scavenging capacities of the fractions from *D. cambodiana and* amounts of total phenolics and total flavonoids in fractions of *D. cambodiana*.

Test materials	DPPH EC_50_	ABTS IC_50_	Superoxideradical (mol α-Tocopherol/g DW)	Total phenolics (GAE g/g extract)	Total flavonoids (RE mg/g extract)
PEF	5.007	1.510	0.221 ± 0.12	4.88 ± 1.35^b^	0
EAF	1.613	0.535	1.028 ±0.53	27.82 ± 1.02^a^	65.70 ± 2.44^a^
BUF	2.441	0.884	0.939 ± 0.46	9.06 ± 0.26^b^	43.59 ± 2.45^a^
AF	4.874	1.100	0.419 ± 0.32	5.02 ± 0.18^b^	15.68 ± 3.14^b^

PEF, EAF, BUF, and AF represent the petroleum ether fraction, ethyl acetate fraction, *n*-butanol fraction and aqueous fraction of *D. cambodiana*, respectively. The EC_50_ values and IC_50_ values of test materials are expressed as mg/mL. Values are means ± SD of three determinations. Different letters in the same column indicate significant difference (P < 0.05).

Superoxide anion radical plays an important role in the formation of other reactive oxygen-species such as hydroxyl radical, hydrogen peroxide, or singlet oxygen in living systems [19], so evaluating the scavenging effects of *D. cambodiana* on superoxide radicals is one of the most important ways to clarify the mechanism of any antioxidant activity. Table 2 shows the superoxide anion radical-scavenging capacities of the fractions of *D. cambodiana* stems. All of the tested extracts exhibited superoxide radical-scavenging effects. Among the fractions, the order of the superoxide anion radical-scavenging capacities were found to be EAF > BUF > AF > PEF.

The overall antioxidant activities of plants are typically due to the presence of phenolic compounds. Generally, the antioxidant activities of phenolic compounds are realized by inactivating lipid free radicals and preventing decomposition of hydroperoxides into free radicals. The amounts of the total phenolics and the total flavonoids in the fractions of *D. cambodiana* stems are shown in Table 2. EAF showed the highest amount of total phenolics and total flavonoids, followed by BUF, AF, and PEF. Many studies have conclusively revealed that the antioxidant activity was related to the amount of total phenolics and total flavonoids [20,21]. At the same time, we could find that this order was similar to that of their antioxidant and radical-scavenging capacities. Results in our study also demonstrated that the extent of antioxidant activity of *D. cambodiana* stems was in accordance with the amounts of phenolics and flavonoids present in this species. The results showed that the ethyl acetate fraction had the strongest DPPH, ABTS free radical, Superoxide radical-scavenging capacities and the highest amount of total phenolics and total flavonoids among the other fractions. We thus choose the ethyl acetate fraction to undergo further chromatography to isolate the sub-fractions containing compounds **1-9**.

### 2.2. Sub-Fractions

Nine compounds were isolated from the ethyl acetate fraction of the *D. cambodiana* stem extract by column chromatography and identified as: 3,4-dihydroxyallyl- benzene (**1**), *p*-hydroxybenzaldehyde (**2**), cambodianol (**3**), (2*S*)-3',7-dihydroxy-4'-methoxy-8-methylflavane (**4**), (2*R*)-4',7-dihydroxy-8-methylflavane (**5**), (±)-4',7-dihydroxy-3'-methoxyflavane (**6**), (2*S*)-4',7-dihydroxyflavane (**7**), 7,4'-dihydroxyhomoisoflavane(**8**) and 4,4'-dihydroxy-2'-methoxychalcone (**9**). Compounds **1** and **2** were categorized as simple phenolics, while **3** − **9** were flavonoids. The antioxidant activities of compounds **1**–**9** isolated from *D. cambodiana* were determined by DPPH radical-scavenging assay, ABTS free radical-scavenging assay and Superoxide anion radical-scavenging assay, and the results were shown in Table 3. 

Compounds **1** and **2** showed higher DPPH radical-scavenging capacity than **4**, **5** or **6**, but their activities were lower than that of ascorbic acid. At the 30 minute reaction timepoint, **3**, **7**, **8** and **9**, had no DPPH radical-scavenging capacity, which might relate to the structure of the compound, the reaction time, and the reaction solvent.

**Table 3:** The DPPH, ABTS^+^ and Superoxide anion radical-scavenging capacities of the isolated compounds from *D. cambodiana*.

Test materials	DPPH EC_50_	ABTS IC_50_	Superoxide radical (mol α-tocopherol/g DW)
Compound **1**	0.087	0.010	4.348 ± 1.74
Compound **2**	0.108	0.012	1.362 ± 0.58
Compound **3**	–	0.138	0.272 ± 0.15
Compound **4**	0.262	0.275	0.087 ± 0.02
Compound **5**	0.535	0.033	–
Compound **6**	0.413	0.040	0.551 ± 0.46
Compound **7**	–	0.021	0.125 ± 0.02
Compound **8**	–	0.022	–
Compound **9**	–	0.046	–
Ascorbic acid	0.069	ND	ND
Trolox^®^	ND	0.089	ND

‘–’ represents the antioxidant capacities of the compounds have no linear relations.

All the compounds examined were found to possess good ABTS free radical scavenging capacities. The ABTS radical-scavenging capacity of compounds **1**, **2**, **7**, **8**, **5**, **6** and **9** was higher than that of the reference compound Trolox^®^. The results showed that some compounds exhibited superoxide radical-scavenging effects. Among these, compounds **1** and **2** showed much higher superoxide anion radical-scavenging capacities, while compounds **5**, **8**, **9** had no superoxide anion radical-scavenging capacities, which might again relate to the structure of these particular compounds.

### 2.3. The Structure-Activity Relationships of Compounds Derived from D. Cambodiana

The three test methods all demonstrated that compound **1** was the most active free radical scavenger. This might relate to the double bond and the number of hydroxyls present in the aromatic ring. The results also support the notion that *ortho*-hydroxyl structures are crucial for the enhanced antioxidant activity because the *ortho*-quinone is easily formed [22,23]. Compound **2** was the second most active free radical scavenger. Aside from hydroxyls, aldehydes might play an important role in the antioxidant activity. The antioxidant activities of compounds **4**–**9** had no evident differences. In addition, compound **1** showed higher inhibitory activity towards *Staphylococcus aureus* and methicillin-resistant *Staphylococcus aureus*, therefore, it has great potential for pharmaceutical applications.

## 3. Experimental

### 3.1. General

Optical density measurements were made with a Shimadzu UV-2550 spectrophotometer (Shimadzu, Kyoto, Japan). 1,1-Diphenyl-2-picrylhydrazyl radical (DPPH·), 2,2'-azinobis-(3-ethylbenzothiazoline-6-sulfonate) (ABTS^+^), 6-hydroxy-2,5,7,8-tetramethylchroman-2-carboxylic acid (Trolox^®^) and ascorbic acid were purchased from Sigma-Aldrich (St. Louis, MO, USA). All other chemicals were of analytical reagent grade and used without any further purification.

### 3.2. Plant Materials

The stems of *D. cambodiana* were collected in Haikou, Hainan Province, China in July 2007, dried immediately and crushed into pieces. The specimen was identified by Associate Professor Dai Zheng-Fu of the Institute of Tropical Bioscience and Biotechnology, Chinese Academy of Tropical Agricultural Sciences, where a voucher specimen (No. 20070701) of *D. cambodiana* was deposited.

### 3.3. Extraction and Isolation of Antioxidant Compounds

The dried and crushed stems of *D. cambodiana* (13.3 kg) were exhaustively extracted three times with 95% ethanol (50 L) at room temperature for three weeks. The ethanol extract was then filtered through absorbent gauze, and the filtrate was concentrated under reduced pressure at 50 °C to remove ethanol. The extract was suspended in H_2_O (2 L) and partitioned with petroleum ether (1 L × 3), and the resulting supernatants were collected and filtered through absorbent gauze, followed by evaporation of the solvent at 50 °C under reduced pressure. The resulting liquid residue was labeled as the petroleum ether fraction. The defatted material remaining after petroleum ether extraction was partitioned successively with ethyl acetate (1 L × 3) and *n*-butanol (1 L × 3). The ethyl acetate and *n*-butanol extracts were separately combined and evaporated to dryness under reduced pressure, while the aqueous layer was lyophilised to dryness. These four fractions were designated as PEF (15.5 g), EAF (145.0 g), BUF (400.0 g), and AF (13.0 g), respectively. The EAF fraction (145.0) was subjected to column chromatography (CC) over silica gel eluted with a mixture of chloroform and methanol (100:1–0:100, v/v) of increasing polarity resulting in six fractions (Fr.1–Fr.6). Repeated CC on silica gel CC eluted with petroleum ether-acetone gradients (10:1–2:1, v/v) and Sephadex LH-20 (CHCl_3_-MeOH, 1:1, v/v), led to the isolation of compounds **1** (116.6 mg), **3** (120.9 mg), **4** (26.3 mg), **5** (13.6 mg), **6** (10.2 mg) and **8** (8.3 mg) from Fr.2 (16.0 g). Fr.3 (24.0 g) yielded compounds **2** (9.8 mg), **7** (50.3 mg) and **9** (177.7 mg) after CC with Sephadex LH-20 (CHCl_3_-MeOH, 1:1, v/v) and further purification with silica gel CC (CHCl_3_-MeOH, 30:1). The structures of these compounds were identified on the basis of physicochemical properties and spectroscopic data [14,15,16].

### 3.4. Assays

#### 3.4.1. DPPH scavenging capacity

The DPPH radical-scavenging capacity was measured using the method of Zhang and Lu [24,25] with some modification. Two milliliter of an ethanol solution of DPPH (0.1 mM) was added to sample fractions (0.1 mL, 25–800 μg/mL) or individual pure compounds (6.25–200 μg/mL) in DMSO at different concentrations. After gentle mixing and 30 min of reaction at room temperature, the absorbances of the resulting solutions were measured at 517 nm. Ascorbic acid was used as the positive control. The DPPH radical-scavenging capacity (%) was calculated using the following equation:

DPPH radical-scavenging capacity (%) = [1 − (A1 − A2) / A0] × 100%

where A_0_ was the absorbance of the control (without sample), A_1_ was the absorbance in the presence of the sample, and A_2_ was the absorbance without DPPH. The EC_50_ valuee, defined as the amount of antioxidant necessary to decrease the initial DPPH˙ concentration by 50%, were calculated from the results. Measurements were calibrated to a standard curve of prepared ascorbic acid (6.25–200 μg/mL) y = 0.0074x − 0.0110, R^2^ = 0.9958.

#### 3.4.2. ABTS free radical scavenging assay

The ABTS free radical scavenging capacity assays were carried using a modified method as described by Fang and Re [26,27]. Potassium persulfate was added to 7 mM of ABTS^+^ and kept for 12–16 h at room temperature in dark. The ABTS^+^ solution was diluted with PBS (potassium phosphate-buffered saline, pH 7.4) to an absorbance of 0.70 ± 0.02 at 734 nm before analysis. ABTS^+^ solution (3.0 mL) was added to sample fractions (0.1 mL, 25–800 μg/mL) or individual pure compounds (6.25–200 μg/mL) in DMSO at different concentrations and mixed by hand for 20 s. The reaction mixture was kept at room temperature for 6 min, and the absorbance was recorded at 734 nm on a Shimadzu UV-2550 spectrophotometer. Trolox^®^ was used as the positive control. The ABTS free radical-scavenging capacity (%) was calculated using the following equation:

ABTS free radical-scavenging capacity (%) = [1 − (A1 − A2) / A0] × 100%

where A_0_ was the absorbance of the control (without sample), A_1_ was the absorbance in the presence of the sample, and A_2_ was the absorbance without ABTS^+^. The IC_50_ values, defined as the amount of antioxidant necessary to decrease the initial ABTS^+^ concentration by 50%, were calculated from the results. Measurements were calibrated to a standard curve of prepared Trolox^®^ (6.25 − 200 *μ*g/mL) y = 0.0050x + 0.0569, R^2^ = 0.9749.

#### 3.4.3. Superoxide radical-scavenging capacity

The superoxide anion radical-scavenging capacities of the sample were tested by the method of Sakanaka *et al.* [28]. Briefly, phosphate buffer solution (1.0 mL, 65.0 mM, pH 7.8), xanthine solution (0.1 mL, 7.5 mM), ydroxylammonium chloride solution (0.1 mL, 10.0 mM), and sample fractions(0.1 mL, 0.1 mg/mL) or individual pure compounds (0.1 mg/mL), redistilled water (0.4 mL) and protein xanthine oxidase solution (0.3 mL, 20.0 μ*g*/mL) were mixed in turns. The mixture was incubated for 20 min at 25 °C, followed by the addition of anhydrous *p*-aminobenzenesulfonic acid (0.5 mL, 19.0 mM) and α-naphthylamine solution (0.5 mL, 1.0%). The reaction mixture was kept at room temperature for 20 min, and the absorbency (A_1_) was tested at 530 nm. The sample was substituted by redistilled water and repeated the procedures mentioned above, the absorbency (A_0_) of the blank was tested. The standard curve for the superoxide anion radical-scavenging capacity was developed using α-tocopherol standard solution (0–1000 *μ*M) y = 0.00004x + 0.00090, R^2^ = 0.9592. The superoxide radical-scavenging capacity was shown with α-tocopherol equivalent antioxidant capacity (mol α-tocopherol/g DW).

#### 3.4.4. Determination of the amount of total phenolics

The amount of total phenolic contents was determined according to the Folin-Ciocalteu method [29] with some modifications. Briefly, sample fractions (1.0 mL) were was mixed with distilled water (9.0 mL) in a 25 mL volumetric flask. Then Folin-Ciocalteu’s phenol reagent (1.0 mL) was added to the mixture which was then shaken. The mixture was kept for 5 min, followed by the addition of 7% Na_2_CO_3_ solution (10 mL). The mixed solution was then diluted to 25 mL with distilled water and mixed thoroughly. After 90 min of reaction at room temperature, the absorbance versus a blank was measured at 750 nm. The standard curve for total phenolics was developed using gallic acid standard solution (0–100 mg/L) y = 99.5670x + 0.0425, R^2^ = 0.9952. The total phenolics in the extract and the fractions were expressed as g gallic acid equivalents (GAE)/g extract. All samples were tested in three times, and the results were averaged.

#### 3.4.5. Determination of the amount of total flavonoids

The amount of total flavonoids was determined using the method described by Jia *et al.* [30]. Briefly, sample fractions or standard solution of rutin (1 mL) was mixed with distilled H_2_O (4 mL) in a 10 mL volumetric flask, followed by the addition of 5% NaNO_2_ solution (0.3 mL). After 5 min, 10% AlCl_3_ solution (0.3 mL) was added. At 6 min, 1 M NaOH solution (2 mL) was added to the mixture. Immediately, distilled H_2_O (2.4 mL) was added to the reaction flask and the contents mixed well. The absorbance versus a blank was measured at 510 nm. Measurements were calibrated to a standard curve of prepared rutin standard solution (0–0.5 mg/L) y = 1.0503x + 0.0176, R^2^ = 0.9941. Total flavonoids of the extracts and the fractions were expressed on an extract weight basis as mg/g rutin equivalents (RE). All samples were analyzed in three replications.

### 3.5. Statistical Analysis

Results were expressed as mean ± standard deviation (S.D.) of replicate solvent extractions and triplicate of assays and analyzed by SAS. One-way analysis of variance (ANOVA) with Tukey’s test was carried out to test significant differences between levels of treatment. Significant levels were defined using the values p < 0.05 and p < 0.01. Pearson correlations between variables were established using SAS.

## 4. Conclusions

All the tested extracts of *D. cambodiana* showed antioxidant and radical-scavenging capacities, and the order of antioxidant and radical-scavenging capacities among the extracts assayed through all the three methods was found to be EAF > BUF > AF > PEF. This order was similar to the total phenolics and total flavonoids contents of the fractions. However, the antioxidant and radical-scavenging capacities of some compounds isolated from the stems of *D. cambodiana* were discordant in the three methods. Therefore, we should use at least two methods to evaluate whether a sample has antioxidant activity. The EAF and compound **1** had the lowest IC_50_ values for DPPH, ABTS radical-scavenging capacities and showed the highest superoxide anion radical-scavenging capacities. The DPPH˙, ABTS^+^ and Superoxide anion radical-scavenging capacities of most compounds are higher than the fractions. The results not only confirmed that these compounds were the main constituents contributing to the antioxidant activities of the ethyl acetate fraction, but also indicated that the extracts and compounds from *D. cambodiana* might be used as natural antioxidants and alternatives to synthetic antioxidants and seem to be applicable in both the health and food industry. In order to better utilize the source of *D. cambodiana*, it was necessary to isolate and identify the components in the *n*-butanol fraction, whose antioxidant capacity was similar to the ethyl acetate fraction.
